# Discussion of relationships among changes of pathological indicators, postoperative lymphedema of the upper limb, and prognosis of patients with breast cancer

**DOI:** 10.1042/BSR20190231

**Published:** 2019-04-16

**Authors:** Xiping Zhang, Binbin Tang, Dehong Zou, Hongjian Yang, Enqi Qiao, Xiangming He, Feijiang Yu

**Affiliations:** 1 Department of Breast Surgery, Zhejiang Cancer Hospital, Hangzhou 310022, Zhejiang Province, China; 2 Second Outpatient Department of Traditional Chinese Internal Medicine, Tongde Hospital of Zhejiang Province, Hangzhou 310012, Zhejiang Province, China; 3 Medical Records Room, Zhejiang Cancer Hospital, Hangzhou 310022, Zhejiang Province, China

**Keywords:** Breast Cancer, Lymphedema of the Upper Limb, Pathology, Prognosis

## Abstract

**Objectives:** The present study aimed to discuss the impacts of changes to pathological indicators of patients with breast cancer upon the incidence of postoperative lymphedema of the upper limb and prognosis. **Methods:** 2597 female patients with breast cancer who received surgical treatment in our hospital were enrolled in the present study to evaluate the incidence of these patients’ postoperative lymphedema of the upper limb. **Results:** For patients with breast cancer, the incidence of postoperative lymphedema of the upper limb was related to T stage of breast cancer, lymph node metastasis, the number of metastatic lymph nodes, pTNM stage, and pathological types of breast cancer (*P*<0.05). Lymph node metastasis was an independent risk factor of lymphedema of the upper limb; lymph node metastasis and Ki-67 expression level were independent factors that impacted pathologic complete response rate of neoadjuvant chemotherapies. Patients’ mortality was correlated to pathological and molecular subtypes, Ki-67 expression level, ER expression level, PR expression level, and pTNM stage (*P*<0.05), among which the pTNM stage, Ki-67 expression level, and PR expression level were independent factors that affected prognosis of patients with breast cancer. **Conclusion:** Patients with lymph node metastasis were more prone to lymphedema of the upper limb, while it was easier for those whose Ki-67 expression level was high and who were not subject to lymph node metastasis to get a pathological complete response after receiving neoadjuvant chemotherapies. The prognosis was poorer among patients whose progesterone receptors were negative and Ki-67 expression levels were high at the advanced pTNM stage.

## Background

Breast cancer is the most common malignancy among women; with the popularization of early diagnosis and development of modern therapies, the survival of patients with breast cancer has been greatly prolonged; meanwhile, patients with breast cancer have higher standards for their life expectancy and living standards [1]. Thus, it is of great significance for patients with breast cancer to clarify their prognosis and improve their living standards. Prognosis of these patients is impacted by numerous factors, among them, the pathological index of breast cancer is most closely related to prognosis. It has an important influence on the timing and therapeutic effect of operation, sensitivity of chemotherapy, long-term recurrence, metastasis, survival rate, and so on [2–4]. These years, pathological indicators of breast cancer have been also discovered to be more or less correlated to the incidence of breast cancer related lymphedema (BCRL) [5–7]. BCRL is one of common postoperative complications of breast cancer and its incidence has become increasingly higher [8]. It seriously has an impact on patients’ living standards because it would cause limb edema, deformity, and upper limb dysfunction [9]. For the time being, people have known something about how pathological indicators of breast cancer are related to BCRL and prognosis, but there is still a lack of in-depth research reports. In the present study, 2597 female patients with breast cancer were investigated from the perspective of their prognosis and incidence of BCRL. Besides, the relationships between BCRL and pathological indicators of breast cancer were analyzed, in hope of providing some help for early prevention and improve prognosis of lymphedema.

## Materials and methods

### Case selection

Female patients with breast cancer who underwent surgical treatment in our hospital from December 2011 to December 2014 were selected. The inclusion criteria were described as follows. Firstly, they were pathologically diagnosed with breast cancer before or after operation. Secondly, the medical records were complete. Thirdly, they were not complicated with other diseases such as pathological changes of lymphatic vessels or veins that would cause limb swelling or induce upper limb lymphedema before operation. The patients who accepted modified radical mastectomy, breast-conserving radical mastectomy, axillary lymph node dissection or combined breast reconstruction operation were included. The present study excluded (1) patients whose clinical data or follow-up data were seriously lost, (2) those with communication barriers, and (3) those complicated with other diseases that could cause edema. After selection, 2597 postoperative female patients with breast cancer were included in the present study. The youngest patient was 23 and the oldest patient was 85, while their mean age was (49.9 ± 10.1) [10]. A total of 456 (17.5%) out of all respondents received neoadjuvant chemotherapies. All patients were operated on by more than a dozen doctors with senior professional titles, who have received standardized training in order to control the consistency rate of axillary lymph node dissection surgery. If axillary sentinel lymph node biopsy results are negative, axillary lymph node dissection is usually not performed.

### Standards for molecular classification

Molecular subtypes were immunohistochemically determined according to the St Gallen 2013 consensus guidelines: Luminal A subtype is ER-positive and/or PR-positive, and Her-2 negative (Ki-67<14%); Luminal B1 subtype is ER-positive and/or PR-positive, and Her-2 negative (Ki-67≧14%); Luminal B2 subtype is ER-positive and/or PR-positive, and Her-2 positive (Ki-67 at any level); Her-2 overexpression (Her-2 positive): ER- and PR-deficient, Her-2 overexpression; Triple negative: ER- and PR-deficient, Her-2 negative.

### Measurements for lymphedema of the upper limb and follow-up observation

Objective measurements were made in combination with patients’ subjective feelings. For objective measurements, limb circumference was measured as follows. Patients’ metacarpophalangeal joints, wrist joints, and elbow joints were measured in about 20 cm, 15 cm, 10 cm, and 5 cm above or below, respectively. Then, all numerical values were added to determine the sum. Provided that the sum of the numerical values of the affected side was 5 cm higher than the opposite side, or any measured part was 2 cm longer than the opposite side, it would be defined as lymphedema. Concerning patients’ subjective feelings, patients were asked to answer the following questions in the form of a questionnaire. They were asked if they discovered swelling on affected upper limbs and whether they felt heavy or numb on the affected upper limbs. If the answer was definite, it meant the existence of upper limb lymphedema [10].

### Data collection and follow-up observation

A questionnaire survey was conducted by phone and outpatient follow-up observation. Patients’ medical records in hospitals were consulted, including age, tumor position, tumor size, number of metastatic lymph nodes, pTMN stage, pathological molecular types (including ER, PR, Her-2, Ki-67, and so on), pathological complete remission rate after neoadjuvant chemoradiotherapy, and mortality. The follow-up observation lasted from 6 to 60 months. To be exact, the patients were visited every three or six months. During the follow-up visit, upper limb lymphedema was found in 277 patients and its morbidity was 10.7%. The death patients were 45 cases, and the death rate was 1.73%.

### Statistical analysis

Data were sorted out and statistically analyzed by SPSS 18.0. A univariate analysis was performed to compare how pathological indicators were correlated to lymphedema and prognosis by Chi-square test (χ^2^) and/or Fisher’s exact probability test. Based on statistical results of the univariate analysis, corresponding pathological indicators were chosen as independent variables for a Logistic regression analysis. The two-tailed statistical significance level was taken to be below 0.05.

## Results

### Clinicopathological data

Among 2597 female patients with breast cancer, there were 1309 patients with cancer on the left breast, 1236 patients with cancer on the right breast, and 52 patients with cancer on both the left and right breasts. Lymph node metastasis was detected in 1096 patients and its rate was 42.2%. There were 2145 patients with less than 3 metastatic lymph nodes (including 3, N1 Group), 267 patients with 4–9 metastatic lymph nodes (N2 Group), and 185 patients with more than 10 (including 10) metastatic lymph nodes (N3 Group). In terms of pTMN stage, 233 (9.0%), 646 (24.9%), 1223 (47.0%), 493 (19.0%), and 2 (0.1%) patients were at Stage 0, Stage I, Stage II, Stage III, and Stage IV, respectively. There were 233 patients with ductal carcinoma *in situ*, 2149 patients with infiltrating ductal carcinoma, and 215 patients with pathological types. The expression level of Ki-67 was low in 819 patients and high in 1644 patients, while data were missing in 134 patients. No information about pathological molecular types was recorded in medical records of 45 patients. Among the remaining patients, patients with Luminal A breast cancer accounted for the highest proportion (30.7%), followed by patients with triple-negative breast cancer (21.9%), overexpressed Her-2 (18.7%) and Luminal B2 breast cancer (8.0%), and Luminal B1 breast cancer (1.5%) successively. A total of 489 Her-2 positive patients with breast cancer rejected undertaking the FISH test, so their molecular types of breast cancer could not be judged.

### Relationships between pathology of breast cancer and lymphedema

#### Relationships among diseased position of breast cancer, pTNM stage, and lymphedema

The incidence of BCRL is unrelated to diseased parts of breast cancer (*P*>0.05), but correlated to T stage of breast cancer (χ^2^ = 10.970, *P* = 0.027), lymph node metastasis (χ^2^ = 21.119, *P* = 0.000), the number of metastatic lymph nodes (χ^2^ = 11.232, *P* = 0.004), and pTNM stage (χ^2^ = 22.095, *P* = 0.002), as shown in Table 1. According to further statistical analysis, the incidence of lymphedema was far lower among patients at the Stage Tis compared with those at stages T1, T2, and T3 (*P*<0.01). The incidence of lymphedema did not significantly differ among other T stages (*P*>0.05). Since there were only a few patients at the Stage T4, the difference in the incidence of lymphedema was not statistically significant for patients of this stage. Concerning lymph node metastasis, the morbidity of lymphedema was significantly higher in N3 Group than that of N1 Group (χ^2^ = 10.914, *P* = 0.001), but there were no significant differences between N1 Group and N2 Group, and N2 Group and N3 Group (*P*>0.05). The statistical results on pTNM stage of breast cancer suggested that the risk of edema was significantly lower in patients of Stage 0 than those of Stage II (χ^2^ = 10.057, *P* = 0.002) and Stage III (χ^2^ = 14.799, *P* = 0.000). The incidence of edema was higher in Stage I patients than Stage II (χ^2^ = 6.003, *P* = 0.014) and Stage III patients (χ^2^ = 11.712, *P* = 0.001). It showed no significant difference among all other pTNM stages (*P*>0.05).

**Table 1:** The relationship of tumor position and pTMN staging to lymphedema

Variables	Assignment	*N*	Lymphedema case (%)	χ^2^	*P*
Part of tumor					
	Double breasts	52	4 (7.7)	0.840	>0.05
	Right breast	1236	135 (10.9)		
	Left breast	1309	138 (10.5)		
T staging				10.970	0.027
	T1	1010	107 (10.6)		
	T2	1198	140 (11.7)		
	T3	152	19 (12.5)		
	T4	4	0 (0.0)		
	Tis	233	11 (4.7)		
Lymph node metastasis				21.119	0.000
	No	1501	123 (8.2)		
	Yes	1096	154 (14.1)		
The number of metastatic lymph nodes				11.232	0.004
	≤3 (N1 Group)	2145	212 (10.0)		
	4–9 (N2 Group)	267	32 (12.0)		
	≥10 (N3 Group)	185	33 (17.8)		
pTMN Staging				22.095	0.002
	0 Stage	233	11 (4.7)		
	I Stage	646	52 (8.0)		
	II Stage	1223	143 (11.7)		
	III Stage	493	71 (14.4)		
	IV Stage	2	0 (0.0)		

Notes: Tis: tumor *in situ*; Similarly hereinafter.

#### Relationships between pathological types of breast cancer and lymphedema

In the present study, breast cancer was pathologically classified into infiltrating ductal carcinoma and ductal carcinoma *in situ*. According to our research results, the incidence of lymphedema was 11.3% for infiltrating ductal carcinoma, and 4.7% for ductal carcinoma *in situ*. The incidence of both carcinomas was statistically significant (χ^2^ = 9.471, *P* = 0.002). However, no significant difference existed in the incidence of edema among pathological molecular subtypes (*P*>0.05), as shown in Table 2. Furthermore, the impacts of tumor size, lymph node metastasis, and preoperative chemotherapy upon the incidence of lymphedema were analyzed for infiltrating ductal carcinoma and ductal carcinoma *in situ*. The results showed that the above factors did not significantly affect the incidence of lymphedema among patients with ductal carcinoma *in situ* (*P*>0.05). In patients with infiltrating ductal carcinoma, the incidence of lymphedema was related to lymph node metastasis (χ^2^ = 14.158, *P* = 0.000), but unrelated to the number of metastatic lymph nodes, tumor size, and receipt of neoadjuvant chemotherapy (*P*>0.05), as shown in Tables 3 and 4.

**Table 2:** The relationship between pathological types and lymphedema

Variables	Assignment	*N*	Lymphedema case (%)	χ^2^	*P*
Pathological types					
	IDC	2149	242 (11.3)	9.471	0.002
	DCIS	233	11 (4.7)		
	Others	215	24 (11.2)		
Pathological molecular types					
	Her-2 positive	476	49 (10.3)	2.104	0.465
	Luminal A	784	83 (10.6)		
	Luminal B1	39	3 (7.7)		
	Luminal B2	204	25 (12.3)		
	Triple negative	560	69 (12.3)		
	Typing failure	489	46 (9.4)		
	Missing data	45	2 (4.4)		

Notes: DCIS: ductal carcinoma *in situ*; IDC: infiltrating ductal carcinoma; Similarly hereinafter.

**Table 3:** Univariate analysis of factors impacting the incidence of IDC

Variables	Assignment	*N*	Lymphedema case (%)	χ^2^	*P*
Lymph node metastasis					
	No	1150	102 (8.9)	14.158	0.000
	Yes	999	140 (14.0)		
The number of metastatic lymph nodes					
	≤3 (N1 Group)	1746	185 (10.6)	5.583	0.054
	4–9 (N2 Group)	241	30 (12.4)		
	≥10 (N3 Group)	162	27 (16.7)		
Tumor size					
	≤2cm	925	100 (10.8)	0.728	0.867
	2–5cm	1083	126 (11.6)		
	>5cm	136	15 (11.0)		
Neoadjuvant chemotherapy					
	No	1755	197 (11.2)	0.143	0.931
	Yes	393	45 (11.5)		

**Table 4:** Univariate analysis of factors impacting the incidence of DCIS

Variables	Assignment	*N*	Lymphedema case (%)	χ^2^	*P*
Lymph node metastasis					
	No	228	11 (4.8)	0.253	1.000
	Yes	5	0 (0.0)		
The number of metastatic lymph nodes					
	≤3 (N1 Group)	230	11 (4.8)	0.151	1.000
	4–9 (N2 Group)	3	0 (0.0)		
	≥10 (N3 Group)	0	0 (0.0)		
Tumor size					
	≤2cm	104	6 (5.8)	1.157	0.763
	2–5cm	111	5 (4.5)		
	>5cm	16	0 (0.0)		
Neoadjuvant chemotherapy					
	No	215	9 (4.2)	1.771	0.183
	Yes	18	2 (11.1)		

#### Multivariate analysis of pathological indicators impacting lymphedema

A multivariate analysis was further performed based on statistical results of the univariate analysis. T stage (Tis, T1, T2, T3, and T4), lymph node metastasis, and pathological molecular subtypes (including Luminal A, Luminal B1, Luminal B2, Her-2 positive, triple negative, and others) were reckoned as independent variables. The results suggested that lymph node metastasis (OR = 1.523, *P* = 0.017) was an independent risk factor of postoperative lymphedema after the treatment of breast cancer, as shown in Table 5.

**Table 5:** Logistic regression results of multiple factors impacting edema

Variables	β	Wald	*P*	OR	OR 95% CI
T Stage			0.129		
Tis				1	
T1 and T2 Stage	0.661	4.095	0.043	1.937	1.021–3.675
T3 and T4 Stage	0.621	2.313	0.128	1.860	0.836–4.140
Lymph node metastasis	0.535	16.352	0.000	1.707	1.317–2.213
Pathological molecular types			0.612		
Luminal A				1	
Luminal B1	−0.342	0.310	0.577	0.710	0.213–2.366
Luminal B2	0.175	0.512	0.474	1.191	0.738–1.923
Her-2 positive	0.050	0.068	0.794	1.052	0.720–1.535
Triple negative	0.189	1.179	0.278	1.208	0.859–1.700
Others	−0.136	0.500	0.480	0.873	0.599–1.272

### Relationships between pathology and prognosis of breast cancer

#### Relationships between pathology and chemotherapeutic effects of breast cancer

In the present study, 456 patients received neoadjuvant chemotherapies, including 34 patients with a pathological complete response, so the PCR (pathological complete remission) rate was 7.5%. According to the results, the PCR rate of neoadjuvant chemotherapies for breast cancer was related to the Ki-67 expression level (χ^2^ = 9.061, *P* = 0.003), lymph node metastasis (χ^2^ = 58.384, *P* = 0.000), and pTNM stage (χ^2^ = 18.772, *P* = 0.000), but unrelated to tumor size, Her-2 expressions, expressions of hormone receptors, attack of triple-negative breast cancer, and pathological molecular subtypes (*P*>0.05), as shown in Table 6. Tumor size, lymph node metastasis, Ki-67 expression level, and PR expression level were deemed as independent variables for a multivariate logistic analysis. After the analysis, it was discovered that lymph node metastasis (OR = 0.015, *P* = 0.000) and Ki-67 expression level (OR = 6.071, *P* = 0.018) were independent factors that impacted PCR (Table 7).

**Table 6:** The relationship between pathological indicators and the efficacy of neoadjuvant chemotherapy

Variables	Assignment	*N*	PCR *n* (%)	χ^2^	*P*
Her-2				0.214	0.644
	Positive	106	9 (8.5)		
	Negative	350	25 (7.1)		
Ki-67				9.061	0.003
	Low expression	133	2 (1.5)		
	High expression	293	28 (9.6)		
	Missing data	30	4 (13.3)		
Triple negative				0.638	0.424
	Yes	121	11 (9.1)		
	No	335	23 (6.9)		
ER				2.600	0.107
	Positive	274	16 (5.8)		
	Negative	182	18 (9.9)		
PR				3.256	0.071
	Positive	268	15 (5.6)		
	Negative	188	19 (10.1)		
Pathological molecular types				4.097	0.393
	Luminal A	85	3 (3.5)		
	Luminal B1	8	0 (0.0)		
	Luminal B2	43	5 (11.6)		
	Her-2 positive	106	9 (8.5)		
	Triple negative	121	11 (9.1)		
	Typing failure	89	6 (6.7)		
	Missing data	4	0 (0.0)		
Tumor size				3.828	0.148
	≤2cm	131	7 (5.3)		
	2–5cm	265	25 (9.4)		
	>5cm	60	2 (3.3)		
Lymph node metastasis				58.384	0.000
	No	166	33 (19.9)		
	Yes	340	1 (0.3)		
pTMN Stage				18.772	0.000
	II Stage	207	23 (11.1)		
	III/IV Stage	183	1 (0.5)		

**Table 7:** Logistic regression results of multiple factors impacting the efficacy of neoadjuvant chemotherapy

Variables	β	Wald	*P*	OR	OR 95% CI
Tumor size			0.155		
>5cm				1	
2–5cm	0.907	1.280	0.258	2.477	0.515–11.925
≤2cm	0.097	0.012	0.911	1.102	0.201–6.055
Lymph node metastasis	−4.210	16.863	0.000	0.015	0.002–0.111
The expression level of PR	0.049	0.015	0.902	1.051	0.477–2.314
The expression level of Ki-67	1.803	5.634	0.018	6.071	1.369–26.915

#### Relationships among pathology of breast cancer, lymph node metastasis, and mortality

According to statistical results, the lymph node metastasis rate was unrelated to pathological molecular subtypes (*P*>0.05) among patients with breast cancer, as shown in Table 8. These patients’ mortality was correlated to their molecular subtypes (χ2 = 10.791, *P* = 0.029). The mortality was the highest among patients with Luminal B1 breast cancer (5.1%), but the lowest (0.8%) among patients with Luminal A breast cancer. After further analysis, the mortality of patients with Luminal A breast cancer was lower than patients with Her-2-positive breast cancer (χ^2^ = 7.694, *P* = 0.006), Luminal B1 breast cancer (χ^2^ = 7.296, *P* = 0.007), and Luminal B2 breast cancer (χ2 = 6.332, *P* = 0.012). The differences in mortality were not statistically significant among other types of breast cancer (*P*>0.05). In addition, the mortality of patients with breast cancer was also related to Ki-67 expression level (χ^2^ = 7.889, *P* = 0.005), ER expression level (χ^2^ = 7.869, *P* = 0.005), PR expression level (χ^2^ = 17.917, *P* = 0.000), and pTNM stage (χ^2^ = 46.669, *P* = 0.000), as shown in Table 9. Concerning pTNM stage, the mortality of stage III and IV patients was significantly higher than that of Stage 0 patients (χ^2^ = 12.692, *P* = 0.000), Stage I (χ^2^ = 23.500, *P* = 0.000), and Stage II patients (χ^2^ = 24.518, *P* = 0.000). The mortality did not significantly differ among all other pTNM stages (*P*>0.05). A multivariate analysis was performed by choosing pTNM stage, Ki-67 expression level, ER expression level, and PR expression level as independent variables. According to the results of this analysis, pTNM stage (*P*<0.01), Ki-67 expression level (*P*<0.05), and PR expression level (*P*<0.01) were independent factors that affected prognosis of patients with breast cancer, as shown in Table 10.

**Table 8:** The relationship between pathological molecular types and lymph node metastasis

Molecular Types	*n/N*	Metastasis rate %	χ^2^	*P*
Her-2 positive	193/476	40.5	3.585	0.465
Luminal A	350/784	44.6		
Luminal B1	15/39	38.5		
Luminal B2	88/204	43.1		
Triple negative	229/560	40.9		

**Table 9:** The relationship between pathological indicators and mortality

Variables	Assignment	*N*	Mortality *n* (%)	χ^2^	*P*
PCR				0.183	1.000
	Yes	34	1 (2.9)		
	No	422	19 (4.5)		
					
Ki-67				7.889	0.005
	Low expression	819	5 (0.6)		
	High expression	1644	35 (2.1)		
	Missing data	134	5 (3.7)		
ER				7.869	0.005
	Positive	1771	22 (1.2)		
	Negative	826	23 (2.8)		
PR				17.917	0.000
	Positive	1744	17 (1.0)		
	Negative	853	28 (3.3)		
Pathological molecular types				10.791	0.029
	Her-2 positive	476	13 (2.7)		
	Luminal A	784	6 (0.8)		
	Luminal B1	39	2 (5.1)		
	Luminal B2	204	6 (2.9)		
	Triple negative	560	11 (2.0)		
pTMN Stage				46.669	0.000
	0 Stage	233	0 (0.0)		
	I Stage	646	4 (0.6)		
	II Stage	1223	15 (1.2)		
	III/IV Stage	495	26 (5.3)		

**Table 10:** Logistic regression results of multiple factors impacting the mortality of breast cancer patients

Variables	β	Wald	*P*	OR	OR 95% CI
pTMN stage			0.000		
III/IV Stage				1	
II Stage	−1.493	20.334	0.000	0.225	0.117–0.430
0/I Stage	−2.479	20.924	0.000	0.084	0.029–0.242
The expression level of Ki-67	0.999	4.168	0.041	2.715	1.041–7.081
The expression level of ER	0.183	0.206	0.650	1.201	0.545–2.648
The expression level of PR	−1.215	8.687	0.003	0.297	0.132–0.666

## Discussions

As one of common postoperative complications among patients with breast cancer, BCRL is seriously impacting mental and physiological health of patients. At present, the genesis of BCRL is generally acknowledged to result from multiple factors [1,11]. In addition, we have discovered in our previous study [10] that BCRL is related to postoperative infection, operative techniques, and extent of lymph node dissection. Current studies about risk factors of BCRL mainly focus on personal and therapeutic factors of patients with breast cancer, including weight, blood pressure, infections in upper limbs, and operative techniques [12–14], whereas the relationships between pathological indicators of breast cancer and BCRL have been rarely discussed. In the present study, pathological indicators of breast cancer were found to be somewhat related to the genesis of BCRL. Compared with Stage T1, T2, and T3 patients, the incidence of lymphedema was significantly lower among patients with Stage Tis breast cancer. The risk of lymphedema was 1.7 times higher among patients with breast cancer and lymph node metastasis than those without lymph node metastasis, and proportional to the number of metastatic lymph nodes. The incidence of lymphedema was 1.78 times higher among patients with 10 or more metastatic lymph nodes than others with 3 or fewer metastatic lymph nodes, and 2.17 times higher than others without lymph node metastasis. As regards pTNM stage, the incidence of lymphedema was higher among Stage II and III patients with breast cancer compared with Stage 0 and I patients. The pathological type of breast cancer was another factor that impacted BCRL. Compared with patients with ductal carcinoma *in situ*, the incidence of lymphedema was 2.4 times higher among those with infiltrating ductal carcinoma, and pathological molecular subtypes of breast cancer were insignificantly correlated to the genesis of BCRL. Thus, it was clear that the genesis of BCRL was related to T stage of breast cancer, lymph node metastasis, the number of metastatic lymph nodes, pTNM stage, and pathological types of tumors, perhaps because normal functions of the lymphatic system were heavily impaired in patients with infiltrating ductal carcinoma or lymph node metastasis or patients at the advanced pTNM stage, and lymphatic reflux was blocked owing to their more severe lymph node metastasis. Besides, the extent of surgical resection was usually wider among this type of patients. Before and after operations, more adjuvant therapies like radiotherapies were needed. All these factors would aggravate damages to the lymphatic system and its peripheral tissues. As a consequence, lymphedema occurred after operations were performed upon the affected upper limbs. In addition, some previous studies [15,16] discovered that lymph node metastasis of breast cancer was an important risk factor that influenced the lymphedema of the upper limb, and the incidence of the lymphedema was far lower among Stage N0 patients than Stage N1 or N2 patients [7].

Breast cancer is heterogeneous, while different pathological indicators of breast cancer usually reflect varying chemotherapeutic sensitivity and prognosis. From the perspective of tumor size, previous report [17] pointed out that primary tumor size of breast cancer was related to chemotherapeutic effects, and the pathological complete response rate would be lower after chemotherapies for tumors that were larger in diameter. Nevertheless, in the present study, tumor size was not found to be significantly correlated to the chemotherapeutic effects. In terms of N stage, it was discovered in the present study that neoadjuvant chemotherapies were much more effective for patients without lymph node metastasis than those with lymph node metastasis, so lymph node metastasis was an independent factor that impacted the effects of neoadjuvant chemotherapies. In terms of the general pTNM stage, both the effects of neoadjuvant chemotherapies and mortality were related to pTNM stage among patients with breast cancer. The PCR rate of Stage III and IV patients was lower than Stage II patients in neoadjuvant chemotherapies, whereas the mortality was much higher in Stage 0, I, and II patients. Moreover, pTNM stage was an independent factor that affected the deaths of patients with breast cancer. The mortality of Stage III and IV patients was 8.8 times higher than that of Stage I patients and 4.4 times higher than that of Stage II patients. This research finding was in accordance with the traditional idea that chemotherapeutic effects and prognosis were poorer in patients at the advanced pTNM stage.

As a critical pathological indicator of patients with breast cancer, Ki-67 is a proliferating cell nuclear antigen that is closely related to the period of mitosis. In the present study, it was found to be an independent factor that affected the effects of neoadjuvant chemotherapies and mortality of patients with breast cancer. Patients whose Ki-67 expression level was high could usually achieve better chemotherapeutic outcomes, whereas the mortality was significantly higher than those with low Ki-67 expression level. The 5-year mortality of patients with a high Ki-67 expression level was 3.5 times higher compared with patients whose Ki-67 expression level was low. The Ki-67 expression level was related to genesis, infiltration, and metastasis of tumors. At present, it is generally believed that Ki-67 expression level is an indicator that reflects poor prognosis of patients with breast cancer [18,19]. Patients whose Ki-67 expression level was high were characterized by active proliferation of tumor cells, fast tumor growth, high aggressiveness, high-grade malignancy, high risk of metastasis, and poorer prognosis. Chemotherapy drugs mostly bring about their effects by inhibiting the proliferation of tumor cells and inducing their apoptosis, so patients with high Ki-67 expressions are usually more sensitive to chemotherapies. As an indicator for predicting the effects of neoadjuvant chemotherapies and prognosis, Ki-67 is worth further research.

According to previous research findings [20,21], pathological molecular subtypes of breast cancer are related to the prognosis to a certain extent. Luminal breast cancer is less sensitive to chemotherapies, whereas hormone-receptor-negative, triple-negative, and Her-2-positive breast cancer is more sensitive to chemotherapies. Although the present study found that PCR rate of hormone-receptor-negative patients was higher in neoadjuvant chemotherapies than that of estrogen-receptor-positive patients. The PCR rate of patients with triple-negative, Her-2-positive, and Luminal B2 breast cancer was higher than those with Luminal A and Luminal B1 breast cancer, whereas these differences were not statistically significant, possibly because only a few patients who received neoadjuvant chemotherapies were included in the present study, where chemotherapeutic regimes were not completely the same as those of previous studies. It was previously reported that [22] molecular subtypes of breast cancer were correlated to lymphatic metastasis, and triple-negative subtype was a crucial factor that impacted long-term metastasis. However, the rate of lymph node metastasis was not discovered to be higher in triple-negative breast cancer in the present study. Thus, the relationships between molecular subtypes and lymphatic metastasis remained to be studied more deeply. The mortality of patients with estrogen-receptor-positive breast cancer was much higher than that of patients whose expression of estrogen receptors was negative, and the mortality of estrogen-dependent Luminal A breast cancer was the lowest among pathological molecular types, which was consistent with the previous reports [23,24]. Hormone-dependent breast cancer usually can benefit from endocrine therapies. In addition, we discovered that PR expression level was an independent factor that impacted death of patients with breast cancer. However, this was not the case for ER. Thus, it meant that PR expression level was more valuable than ER for predicting mortality risks of patients.

At present, it has been pointed in some studies that prognosis is more favorable among patients who receive neoadjuvant chemotherapies [25,26]. However, objections were raised in many reports, where it was proposed that overall survival of patients who received neoadjuvant chemotherapies was not significantly prolonged [27,28]. Alfredo and some other scholars [29] discovered after a meta-analysis of 29 clinical trials that it was impossible to predict long-term survival of patients with breast cancer by evaluating effects of neoadjuvant chemotherapies with PCR. Hence, it was inadvisable to assess the therapeutic effects of systemic therapies for breast cancer by treating PCR as a clinical endpoint. Furthermore, it was discovered in the present study that the mortality of patients with breast cancer was not significantly related to PCR at all. Nonetheless, the value of PCR for predicting prognosis remains to be further investigated more deeply.

Above all, pathological indicators of breast cancer are correlated to both prognosis of related patients and BCRL. Lymph node metastasis is most closely associated with BCRL, while pTNM stage and PR expression level have the closest connections with Ki-67 expression level and prognosis. By analyzing pathological indicators of patients with breast cancer, it is helpful for us to measure these patients’ risks of BCRL and intervene with the risks as early as possible. It is also favorable for us to understand prognosis of breast cancer and perform individualized therapies on patients.

## Conclusions

BCRL is one of common postoperative complications of breast cancer and its incidence has become increasingly higher. It seriously has an impact on patients’ living standards because it would cause limb edema, deformity, and upper limb dysfunction [9]. For the time being, people have known something about how pathological indicators of breast cancer are related to BCRL and prognosis, but there is still a lack of in-depth research reports. In the present study, 2597 female patients with breast cancer were investigated from the perspective of their prognosis and incidence of BCRL. Besides, the relationships between BCRL and pathological indicators of breast cancer were analyzed, in hope of providing some help for early prevention and prognosis of lymphedema. We found that pathological indicators of breast cancer are correlated to both prognosis of related patients and BCRL. Lymph node metastasis is most closely associated with BCRL, while pTNM stage and PR expression level have the closest connections with Ki-67 expression level and prognosis. By analyzing pathological indicators of patients with breast cancer, it is helpful for us to measure these patients’ risks of BCRL and intervene with the risks as early as possible. It is also favorable for us to understand prognosis of breast cancer and perform individualized therapies on patients.

## Availability of data and material

The corresponding author Z.X. has the related data.

## Abbreviations

BCRL: Breast cancer related lymphedema
PCR: Pathological complete remission

## References

[1] Michael S., Charikleia S. and Konstantinos K. (2011) Lymphedema and breast cancer: a review of the literature. Breast Cancer 18, 174–180 10.1007/s12282-010-0246-1 21331463

[2] Goorts B., van Nijnatten T.J., de Munck L., Moossdorff M., Heuts E.M., de Boer M. (2017) Clinical tumor stage is the most important predictor of pathological complete response rate after neoadjuvant chemotherapy in breast cancer patients. Breast Cancer Res. Treat. 163, 83–91 10.1007/s10549-017-4155-2 28205044 PMC5387027

[3] Wang G.S., Zhu H. and Bi S.J. (2012) Pathological features and prognosis of different molecular subtypes of breast cancer. Mol. Med. Rep. 6, 779–782 10.3892/mmr.2012.981 22797840

[4] Zhao Y., Dong X., Li R., Ma X., Song J., Li Y. (2015) Evaluation of the pathological response and prognosis following neoadjuvant chemotherapy in molecular subtypes of breast cancer. Onco. Targets Ther. 8, 1511–1521 26150728 10.2147/OTT.S83243 PMC4480585

[5] Krok-Schoen J.L., Oliveri J.M., Kurta M.L. and Paskett E.D. (2015) Breast cancer-related lymphedema: risk factors, prevention, diagnosis and treatment. Breast Cancer Management 4, 41–51 10.2217/bmt.14.51

[6] Zhu Y.Q., Xie Y.H., Liu F.H., Guo Q., Shen P.P. and Tian Y. (2014) Systemic analysis on risk factors for breast cancer related lymphedema. Asian Pac. J. Cancer Prev. 15, 6535–6541 10.7314/APJCP.2014.15.16.6535 25169483

[7] Shah C., Wilkinson J.B., Baschnagel A., Ghilezan M., Riutta J., Dekhne N. (2012) Factors associated with the development of breast cancer-related lymphedema after whole-breast irradiation. Int. J. Radiat. Oncol. Biol. Phys. 83, 1095–1100 10.1016/j.ijrobp.2011.09.058 22099041

[8] Disipio T., Rye S., Newman B. and Hayes S. (2013) Incidence of unilateral arm lymphoedema after breast cancer: a systematic review and meta-analysis. Lancet Oncol. 14, 500–515 10.1016/S1470-2045(13)70076-7 23540561

[9] Ugur S., Arıcı C., Yaprak M., Mescı A., Arıcı G.A., Dolay K. (2013) Risk factors of breast cancer-related lymphedema. Lymphat. Res. Biol. 11, 72–75 10.1089/lrb.2013.0004 23772716 PMC3685313

[10] Zhang X., He X., Tang B., Yang H., Ding X., Yu Y. (2017) Risk factors of lymphedema on affected side of upper limb after breast cancer surgery–report from a single center of China. Int. J. Clin. Exp. Med. 10, 1592–1601

[11] Dominick S.A., Madlensky L., Natarajan L. and Pierce J.P. (2013) Risk factors associated with breast cancer-related lymphedema in the WHEL Study. J. Cancer Surviv. 7, 115–123 10.1007/s11764-012-0251-9 23212606 PMC3568206

[12] Tsai R.J., Dennis L.K., Lynch C.F., Snetselaar L.G., Zamba G.K. and Scott-Conner C. (2009) The risk of developing arm lymphedema among breast cancer survivors: a meta-analysis of treatment factors. Ann. Surg. Oncol. 16, 1959–1972 10.1245/s10434-009-0452-2 19365624

[13] Schmitz K.H., Ahmed R.L., Troxel A.B., Cheville A., Lewis-Grant L., Smith R. (2010) Weight lifting for women at risk for breast cancer-related lymphedema: a randomized trial. JAMA 304, 2699 10.1001/jama.2010.1837 21148134

[14] Rebegea L., Firescu D., Dumitru M. and Anghel R. (2015) The incidence and risk factors for occurrence of arm lymphedema after treatment of breast cancer. Chirurgia (Bucur) 110, 33 25800313

[15] Ahmed R.L., Schmitz K.H., Prizment A.E. and Folsom A.R. (2011) Risk factors for lymphedema in breast cancer survivors, the Iowa Women’s Health Study. Breast Cancer Res. Treat. 130, 981–991 10.1007/s10549-011-1667-z 21761159 PMC4091732

[16] Soyder A., Taştaban E., Özbaş S., Boylu Ş and Özgün H. (2014) Frequency of early-stage lymphedema and risk factors in postoperative patients with breast cancer. Meme Sagligi Dergisi 10, 92–97 28331651 10.5152/tjbh.2014.1973 PMC5351476

[17] Bonadonna G., Valagussa P., Zucali R. and Salvadori B. (1995) Primary chemotherapy in surgically resectable breast cancer. CA Cancer J. Clin. 45, 227 10.3322/canjclin.45.4.227 7600279

[18] Masuda H., Masuda N., Kodama Y. (2011) Predictive factors for the effectiveness of neoadjuvant chemotherapy and prognosis in triple-negative breast cancer patients. Cancer Chemother. Pharmacol. 67, 911–917 20593180 10.1007/s00280-010-1371-4

[19] Yerushalmi R., Woods R., Ravdin P.M., Hayes M.M. and Gelmon K.A. (2010) Ki67 in breast cancer: prognostic and predictive potential. Lancet Oncol. 11, 174 10.1016/S1470-2045(09)70262-1 20152769

[20] Kim S.I., Sohn J., Koo J.S., Park S.H., Park H.S. and Park B.W. (2010) Subtypes and tumor response to neoadjuvant chemotherapy in patients with locally advanced breast cancer. Oncology 79, 324 10.1159/000322192 21430399

[21] Luangdilok S., Samarnthai N. and Korphaisarn K. (2014) Association between pathological complete response and outcome following neoadjuvant chemotherapy in locally advanced breast cancer patients. J. Breast Cancer 17, 376–385 10.4048/jbc.2014.17.4.376 25548587 PMC4278058

[22] Chacón R.D. and Costanzo M.V. (2010) Triple-negative breast cancer. Breast Cancer Res. Bcr 24, S3 10.1186/bcr2574 PMC2972557 21050424

[23] Bai G., Zhang J.Q. and Yang M. (2012) Comparative analysis of clinicopathologic characteristics and prognosis of Luminal A with Luminal B breast cancer. J. Practical Oncol. 27, 55–59

[24] Dvorkin-Gheva A. and Hassell J.A. (2014) Identification of a novel luminal molecular subtype of breast cancer. PLoS One 9, e103514 10.1371/journal.pone.0103514 25076125 PMC4116208

[25] Hirano A., Shimizu T., Kamimura M., Ogura K., Kim N., Setoguchi Y. (2012) Significance of neoadjuvant chemotherapy for breast cancer; prediction of prognosis according to pathological response. Nihon Gekakei Rengo Gakkaishi 37, 210–213 10.4030/jjcs.37.210

[26] Viale G. (2013) Characterization and clinical impact of residual disease after neoadjuvant chemotherapy. Breast 22, S88–S91 10.1016/j.breast.2013.07.016 24074800

[27] Shintia C., Endang H. and Diani K. (2016) Assessment of pathological response to neoadjuvant chemotherapy in locally advanced breast cancer using the Miller-Payne system and TUNEL. Malays. J. Pathol. 38, 25–32 27126661

[28] Kuerer H.M., Yang W.T. and Krishnamurthy S. (2016) Comment on diagnosis of pathological complete response to neoadjuvant chemotherapy in breast cancer by minimal invasive biopsy techniques. Br. J. Cancer 114, e3 10.1038/bjc.2015.475 27092783 PMC4865962

[29] Berruti A., Amoroso V., Gallo F., Bertaglia V., Simoncini E., Pedersini R. (2014) Pathologic complete response as a potential surrogate for the clinical outcome in patients with breast cancer after neoadjuvant therapy: a meta-regression of 29 randomized prospective studies. J. Clin. Oncol. Off. J. Am. Soc. Clin. Oncol. 32, 3883–3891 10.1200/JCO.2014.55.2836 25349292

