# Correction: Discussion of relationships among changes of pathological indicators, postoperative lymphedema of the upper limb and prognosis of breast cancer patients

**DOI:** 10.1042/BSR-20190231_COR

**Published:** 2019-05-15

***Bioscience Reports* (2019) 39(4), BSR20190231; https://doi.org/10.1042/BSR20190231**

After detailed discussion, the authors of the published article have decided to change the order of their authorship listing. The agreed authorship listing is as follows:

Dehong Zou^1^, Binbin Tang^2^, Xiping Zhang^1^, Hongjian Yang^1^, Enqi Qiao^1^, Xiangming He^1^ and Feijiang Yu^3^

^1^Department of Breast Surgery, Zhejiang Cancer Hospital, Hangzhou 310022, Zhejiang Province, China; ^2^Second Outpatient Department of Traditional Chinese Internal Medicine, Tongde Hospital of Zhejiang Province, Hangzhou 310012, Zhejiang Province, China; ^3^Medical Records Room, Zhejiang Cancer Hospital, Hangzhou 310022, Zhejiang Province, China

In addition, Dehong Zou, Binbin Tang and Xiping Zhang should no longer be considered co-first authors.

